# New Records of Aphid Fauna in Turkey

**DOI:** 10.1673/031.010.0501

**Published:** 2010-02-25

**Authors:** Azize Toper Kaygin, Gazi Gorur, Figen Cota

**Affiliations:** ^1^ Bartin University, Forestry Faculty, Department of Forest Engineering, Forest Entomology and Protection, 74100, Bartin, Turkey; ^2^ Nigde University, Science and Arts Faculty, Department of Biology, 51100, Nigde, Turkey

**Keywords:** Aphidoidea, Bartin, *Ceruraphis viburnicola*, *Dysaphis apiifolia*, *Macrosiphum mordvilkoi*, *Petroselinum*, *Rosa*, *Viburnum*

## Abstract

Three aphid species were identified as new records for Turkey aphid fauna from Bartin province. These species are *Ceruraphis viburnicola* (Gillette) (Hemiptera: Aphididae), *Dysaphis apiifolia* (Theobald) (Hemiptera: Aphididae) and *Macrosiphum mordvilkoi* Miyazaki (Hemiptera: Aphididae). These records increase the recorded aphid-fauna of Turkey to 433 species.

## Introduction

Studies of the Turkish aphid fauna were limited until the last decade. Although preliminary studies were done at the beginning of the 1900s, most of these studies were carried out by foreign researchers and were only focused on very small parts of Turkey (Trotter 1903; Fahringer 1922). Çanakcioğlu (1975) reviewed previous studies and listed 258 species. Tuatay (1988, 1991, 1993) added about 30 species as new records. Duzgunes et al. (1982) reported three additional species. Recently, many more studies have organized and added more than 40 new records (Canakcioglu and Toper 1999; Kaygin and Canakcioglu 2003; Toros et al. 2002; Toros et al. 2003; Gorur 2002, 2004; Ozdemir et al. 2005; Asian and Uygun 2005). Remaudière et al. (2006) revised studies conducted on Turkey aphid fauna and listed about 417 species, despite some controversies. Akyurek (2006) and Kaygin et al. (2008) added 11 new records.

## Methods and Materials

This study was conducted between 2005 and 2006. Aphid species were collected in the field from their host plants. The study area was located in the Western Black Sea Region of Turkey, where few detailed studies have been carried out.

Collection and preparation of samples were been done according to the principles of Hille Ris Lambers (1950) and Martin (1983). Species were identified according to Bodenheimer and Swirski (1957), Canakcioglu (1975), and Blackman and Eastop (1994, 2006). Systematic knowledge, host plants, and synonyms of determined species were taken from Blackman and Eastop (1994, 2006) and Remaudiere and Remaudiere (1997). The taxonomic status of the species was checked according to the recent update of Fauna Europaea 1.1 (www.faunaeur.org). Voucher specimens were deposited at the Entomology Department of Forestry Faculty in Bartin and the Department of Biology in Niğde.

## Result

*Ceruraphis viburnicola* (Gillette) (Hemiptera: Aphididae), *Dysaphis apiifolia* (Theobald) (Hemiptera: Aphididae) and *Macrosiphum mordvilkoi* Miyazaki (Hemiptera: Aphididae) were new records for Turkey aphid fauna. All new recorded species belonged to the Aphidinae subfamily and Macrosiphini tribe.

**Ceruraphis Borner, 1926  *Ceruraphis viburnicola* (Gillette, 1909)  *NeoCeruraphis viburnicola* (Gillette, 1909)**
Apterous individuals were collected on *Viburnum* sp. at Bartin on 29 July 2005. The species was also collected on *Viburnum* sp. at Nigde province, which is located at the Inner Anatolia of Turkey, on 23 June 2005. They mainly feed on shoots, undersides of the leaves and flowers, such as the snowball flowers of *Viburnum opulus*. They heavily colonize young leaves and flowers and are attended by ants. Their primary host is *V. opulus*, but a secondary host is as yet unknown. Previously, it has been recorded in Canada and the USA (Maw et al. 2000; www.faunaeur.org).

***Dysaphis* Börner, 1931  *Dysaphis apiifolia* (Theobald, 1923)  *Aphis ferrugineastriata* Essig, 1938**
Apterous individuals found on *Petroselinum* sp. at Hendekyanı (Central Bartin), 22 May 2006. There were dense colonies at the leaf bases that were attended by a lot of ants. This aphid is yellowish-grey to greenish-grey and dusted with wax. They typically colonize heavily the leaf bases of celery and parsley, and become serious pests of these host plants (Blackman and Eastop 2006). They mainly feed on *Apium graveolensis*, *Foeniculum vulgare*, *Petroselinum sativum*, and various *Umbelliferae* species.
  They are essentially distributed in the Afrotropical region, Australian region, East Palaearctic, Near East, Nearctic region, Neotropical region and North Africa (www.faunaeur.org 2007).

**Figure 1:**
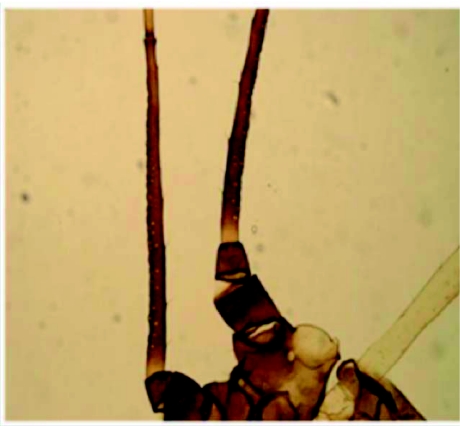
Position of rhinaria on the third antennal segment of *Macrosiphum mordvilkoi* High quality figures are available online.

***Macrosiphum* Passerini, 1860  *Macrosiphum mordvilkoi* Miyazaki, 1968  *Macrosiphum mulgedifolii* Tashev 1967**
Both apterae and alatae forms were collected on *Rosa* spp. at the gardens of Ulus (vicinity of Bartin) and Bartin Forest Enterprise, 31 March 2006. They were abundant in the study area and densely colonized the host plant. The species was identified from apterous individuals. This species was easily separated from other *Macrosiphum* species because of their black front head and antennal segment III with rhinaria extending over most of the length of segment (Figure 1).
  They are distributed in Japan, Korea and eastern Russia (Blackman and Eastop 2006).

## Discussion

Recent studies have shown that aphid species listed for Turkey's aphid fauna do not sufficiently indicate the real number, as there has been much information accumulated during the last decades. These arguments also are supported when Turkey is compared with its neighbours that are located in similar geographic areas in terms of richness of flora, geographic variability, geographic location, agricultural landscape, etc. For example, Tsitsipis et al. (2007) reviewed the known Greek aphid fauna comprising 301 species. Comparatively, the study of the fauna of some countries isn't greater, with about 340 species in Iran and only 167 species in Lebanon and Syria (Remaudière et al. 2006).

The results presented have added three new species to Turkey aphid fauna, so now the Turkey aphid fauna has at least 433 species. Thus, it can be expected that with further research, in different parts of the country, the recorded Turkish aphid fauna will be considerably increased.
